# First in Human Feasibility Study of an Insulin Patch Pump Combined With CGM-Insulin Delivery Cannula

**DOI:** 10.1177/19322968261436412

**Published:** 2026-04-09

**Authors:** Hannah Cunningham, Hanna Jones, Steve Flint, Emma Netzer, Ralph Dutt-Ballerstadt, Peter Eckenberg, Varuni Obeyesekere, Katrin Ellen Brown, Catriona Sims, Solomon Reid, Huan-Ping Wu, Thomas Seidl, Marcel Both, Adrian Auderset, Daniel Eymann, Tamara Lodico, Michael Schoemaker, Yee Wen Kong, David Norman O’Neal

**Affiliations:** 1 Department of Medicine, University of Melbourne, Melbourne, VIC, Australia; 2 University of Melbourne Department of Medicine, St Vincent’s Hospital Melbourne, Fitzroy, VIC, Australia; 3 Pacific Diabetes Technologies, Portland, OR, USA; 4 Pharmasens AG, Biel/Bienne, Switzerland; 5 Michael Schoemaker Consulting, Hassloch, Germany; 6 Australian Centre for Accelerating Diabetes Care, Melbourne, VIC, Australia; 7 Baker Heart and Diabetes Institute, Melbourne, VIC, Australia

**Keywords:** patch pump, continuous glucose monitoring, continuous glucose monitor infusion set, feasibility study

## Abstract

**Aims:**

Combining a patch pump (PP) with a single-insertion glucose-sensing cannula (continuous glucose monitor with an insulin set [CGM-IS]) may reduce user burden compared with tethered insulin pumps or multiple daily injections. We aimed to determine feasibility of a combined device.

**Methods:**

This feasibility study evaluated a PP with a CGM-IS (PP-CGM-IS) with manual bolus insulin dosing in T1D adults with insulin pump and CGM experience. The pilot phase involved 3 participants undergoing a mixed meal test (MMT) for 12 hours. In the second phase, 15 participants were studied for 72 hours, including day 1 and day 3 MMTs and one supervised free-living day at a hotel. Primary outcome was accuracy of the CGM device, assessed by mean absolute relative difference (MARD). Secondary outcomes included number of insulin delivery failures and device survival duration.

**Results:**

Twenty-six devices were inserted in 18 participants. Mean age was 51 years, 14 were female. Nine device (sensor) failures occurred immediately post-insertion requiring re-insertion. Mean absolute relative difference against Yellow Springs Instruments (YSI) in 17 devices was 11.6%. Consensus error grid analysis against YSI analyser showed 83.16% of readings fell within zone A and 100% within zones A and B. Mean glucose, time in range, and total daily insulin dose on day 2 of the main phase compared with baseline did not suggest compromise of insulin delivery. There were no serious adverse events.

**Conclusion:**

This first-in-human study confirms feasibility of integrating CGM-IS with PP. A PP-CGM-IS system warrants further research, including improved insertion process, greater sensor accuracy and ultimately real-time algorithm implementation.

## Introduction

Continuous glucose monitors (CGMs) and insulin pumps while improving glycaemia, may be associated with an added psychological and physical burden.^1^ It is important for healthcare professionals to contextualise the glycaemic benefits within that of the lived experience associated with the use of these wearable devices. Those using CGMs and insulin pumps need to insert two different devices (a CGM sensor and an insulin infusion cannula) subcutaneously each requiring replacement at different intervals. A common issue raised by people with type 1 diabetes (T1D) using these devices is wear related, and barriers included the need to use infusion sets and the need to have a separate CGM sensor attached to the body.^1^ In addition to the subjective burden associated with wearing two devices, there are also health economic and environmental implications resulting from greater waste.

To minimise device complexity, reduce the number of insertion sites, and enhance patient convenience, efforts to integrate a CGM and insulin delivery cannula have been explored.^2-6^ There have been two main approaches to development. The first approach involves integrating a commercially available glucose sensor with a commercially available insulin infusion cannula. Tschaikner et al^4^ evaluated this approach by combining a Dexcom G4 sensor with a standard Medtronic insulin infusion set and cannula, such that the sensing tip lay distal to insulin delivery. They reported a mean absolute relative difference (MARD) of approximately 13%, comparable with a control sensor worn >100 mm away from the investigational device (13.9%).

The second approach involves co-development of a novel glucose sensor with a novel insulin cannula combined within a single device platform. Rumpler et al^3^ described an integrated prototype system with a median absolute relative difference (ARD) of 22.5%. In contrast, commercially available sensors at the time (eg, Dexcom G4) had MARD values of approximately 13%.^7^ More recently, studies evaluating the SynerG device combining CGM with an insulin set (CGM-IS),^5,6^ suggested that neither insulin delivery nor glucose sensor accuracy was compromised during a three-day wear, with an aggregated MARD of 9.0%.^5^ Our prior research indicated significant promise with this approach and the potential for the CGM-IS to be integrated within an insulin patch pump (PP).

Many people living with diabetes prefer the form factor of a PP to a tethered device.^8^ In addition, the tubing associated with tethered pumps is subject to occlusions, kinks and accidental pull outs that interrupt insulin delivery, especially with extended wear sets.^9,10^ Co-location of a CGM system with an insulin infusion set may reduce device burden further, especially if it is integrated with a PP (PP-CGM-IS). Furthermore, a PP-CGM-IS by eliminating the need for digital communication between the CGM and insulin pump may improve communication between these elements minimising signal drop, which would have benefits for automated insulin delivery (AID)-enabled devices.^11^

To our knowledge, this is the first in human study exploring the feasibility of integrating a novel CGM-IS and a novel PP as a single device (PP-CGM-IS).

## Methods

This was a single-centre (St Vincent’s Hospital Melbourne), first in human, single-arm, non-randomised study to explore the feasibility of integrating a CGM-IS with a novel PP. The study consisted of two phases, an initial pilot phase studying three participants wearing the investigational device for 10 to 12 hours and participating in a meal test. The second phase studying 15 participants wearing the investigational device for 72 hours, with the inclusion of two meal tests separated by one free-living day. It was approved by a Human Research Ethics Committee (St Vincent’s Hospital Melbourne HREC reference: 278/24) and the trial was registered (ACTRN12625000244404).

### Participants

Insulin pump and CGM experienced adult patients aged 18 to 75 years with T1D duration of at least six months with a glycated haemoglobin (HbA1c) <10.0% willing and able to meet the protocol requirements were recruited. Participants with a history of recurrent infusion site failure, skin reactions and infections or any other major medical or psychiatric condition precluding their involvement in a clinical trial, including pregnant women or those contemplating pregnancy, were excluded.

### Study Protocol

Following written informed consent, all participants were provided with and educated in the use of a study blood glucose meter (BGM): Ascensia Contour Next meter (Ascensia Diabetes Care, Baulkham Hills, AU) and a Dexcom G6 sensor (Dexcom, San Diego, California) inserted into the abdomen or flank. These were used to guide glycaemic treatment decisions.

#### Pilot phase and meal test protocol

**Figure 1.**
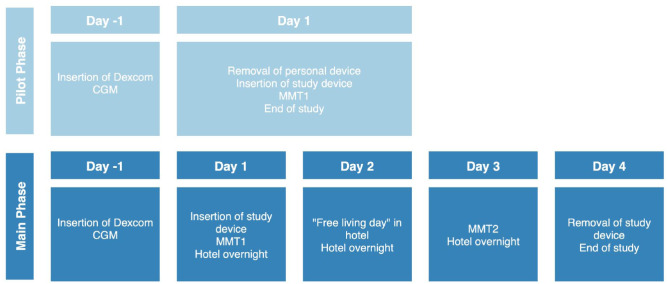
Study protocol.

Participants attended the clinical trials centre at 7:00 am on day 1 of the study after an overnight fast and inserting the Dexcom sensor the day prior to ensure appropriate warm-up time. Upon arrival, the participants’ own insulin pump was disconnected and the investigational PP-CGM-IS was inserted into the abdomen. Insulin delivery with the PP-CGM-IS was not automated and insulin was delivered according to preset basal rates and insulin to carbohydrate ratios (ICRs). After insertion, warm-up and calibration of the CGM-IS sensor, the meal test began. Venous blood samples for glucose measurements were drawn every 15 minutes for one hour. Once four readings were obtained, participants ate a standardised breakfast of 60 g carbohydrate with a pre-meal bolus according to their ICR, 15 minutes prior to eating. Blood samples were collected at 15-minute intervals for an additional four hours. Following completion of the meal test, participants in the pilot phase had the investigational device removed, their own insulin pump was reconnected, and they departed the clinical trials centre (see Figure 1).

#### Main phase

Participants in the main phase attended the clinical trials centre and undertook a meal test as per the pilot phase protocol. They then transitioned to a nearby hotel with medical supervision for the hotel stage of the study. On day 2, participants were instructed to remain at the hotel and refrain from engaging in vigorous activity and consuming alcohol. All meal and correction boluses were administered by trained study staff.

On day 3, participants returned to the clinical trials centre to perform the second standardised meal test, performed as per the day 1 protocol, and returned to the hotel after completion.

On the morning of day 4, the investigational device was removed, and the participants own insulin pump was reconnected. The site was inspected and assessed using the Draize scale, along with clinical photographs of the insertion site.

### Investigational Device

**Figure 2.**
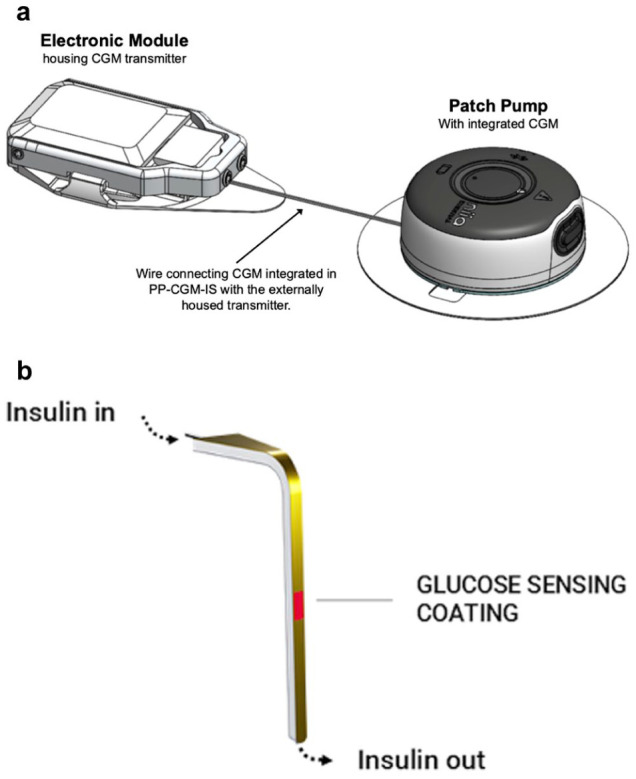
Investigational device. (a) Pharmasens niia essential insulin patch pump with integrated Pacific Diabetes Technology SynerGTM CGM-IS. (b) CGM-IS schematic.

Study participants wore an investigational PP-CGM-IS consisting of a Pharmasens niia essential PP integrated with a Pacific Diabetes Technologies (PDT) SynerG CGM-IS to deliver insulin (Figure 2). The SynerG CGM sensor was fully integrated into the PP; however, the CGM Electronic Module sat external and in close proximity to the pump, connected by a short a wire. This approach makes it possible to investigate the mutual influence of insulin delivery near the CGM sensor in the subcutaneous tissue without the time and financial expenditure involved in fully integrating the sensor electronics into the PP. Full integration can be carried out as part of product development once feasibility has been demonstrated.

The PP-CGM-IS was inserted into the subcutaneous tissue of the abdomen, using a dedicated insertion tool, so that the cannula sat 6 mm below the surface. One fingerprick blood glucose (BG) measurement seven minutes after insertion for calibration was required. Insulin dosing was manually determined for the duration of the study using individualised basal rates, ICRs, and sensitivity factors for each participant. The insulin pump was operated by trained study staff for the duration of the study.

### Biochemical Analysis

During meal tests, blood samples were taken as described above. Capillary blood was used for glucose readings using the study BGM. Plasma glucose concentrations were also measured using a glucose analyser (YSI 2300 STAT Plus Glucose Analyzer YSI Life Sciences, Yellow Springs, Ohio).

### Data Analysis

The primary endpoint was the accuracy of the CGM device, assessed by MARD for YSI readings from 40 to 400 mg/dL. Secondary endpoints for the assessment of CGM-IS sensor accuracy were evaluated by percentage of data points that fall within A&B of the consensus error grid (CEG). The method to calculate glucose estimates was derived retrospectively.

Secondary analyses pertaining to PP-CGM-IS insulin delivery compared mean total daily insulin dose (TDD), time in range (TIR) and mean glucose levels during run-in, 48 hours prior to commencement of the study, against day 2 while wearing the investigational device using paired Student’s *t* test. During the run-in period, participants utilised their usual insulin pumps, insulin infusion sets, and pump settings as well as CGM systems, as per their standard diabetes care, of which one participant used manual insulin dosing. Mean BG levels and time in range (TIR) were calculated based on the data from the commercially available personal CGM systems. Other secondary endpoints included insulin time to maximum bolus and number of insulin delivery failures. Insulin delivery failures were defined by a meter blood glucose (BG) >250 mg/dL, which could not be decreased by 50 mg/dL within one hour of a correction bolus; any meter BG >250 mg/dL and serum ketones >0.6 mmol/L in the absence of infection at the infusion site; an infection at the infusion site; an occurrence of an insulin pump occlusion alarm signal.

Technical endpoints included failure rate of devices and system survival duration. Device survival was defined by the time to failure of either the glucose sensing or insulin delivery. If a device failed within the first 48 hours, it was replaced. After 48 hours, replacement was at the investigator’s discretion. Safety endpoints included the number, description and seriousness of adverse events and adverse device effects.

## Results

**Figure 3.**
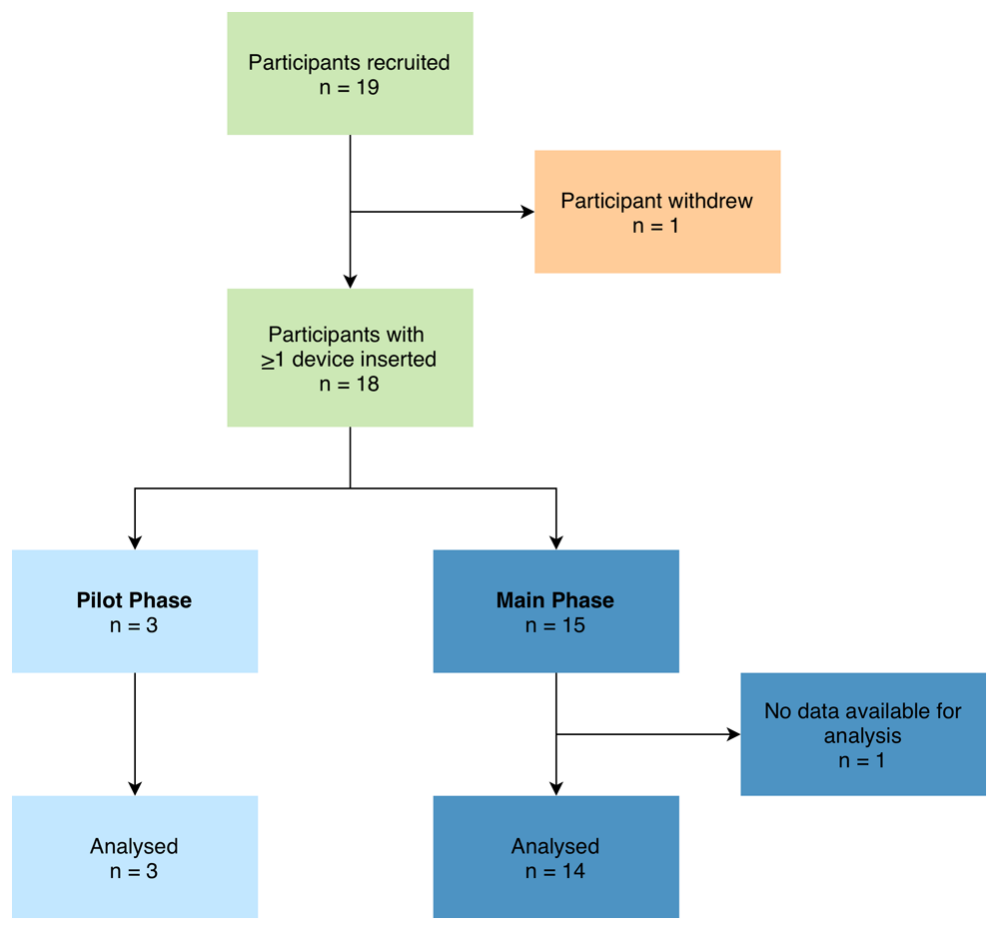
Participant flow chart.

Nineteen participants in total were consented. The first participant was enrolled on 19 March 2025, and the final participant completed the study on May 8, 2025. All three who consented for the pilot phase completed the study. Of the 16 participants consented for the main phase, one withdrew (participant 17) before any study procedure took place and 15 of the participants assigned to the main phase completed the study (Figure 3). Baseline characteristics for all 18 participants (three for the pilot phase and 15 for the main phase) who had at least one sensor inserted are shown in Table 1.

**Table 1.** Baseline Characteristics.

	(n = 18)
Age	51.1 ± 17.3
Female	14 (77.8)
T1D duration (years)	29.9 ± 13.8
Baseline pump type	
Medtronic 780G	11 (61.1)
T: Slim x2	5 (27.8)
YpsoPump	2 (11.1)
CSII duration	12.2 ± 6.9
BMI	29.5 ± 5.0
HbA1c (%)	6.8 ± 0.6
HbA1c (mmol/mol)	50.7 ± 5.9

Categorical variables expressed as n (%) and continuous variables expressed as mean (SD).

There was no safety issue identified during the pilot phase and the study continued to the main phase without requiring any alterations to the protocol or investigational device.

### Device Survival

A total of 38 devices were utilised during the study. On receipt and review of the devices prior to the start of the study and their insertion, a total of 12 systems were excluded from use. Ten of these systems had needle blockages. In two other devices, the cover needed to be replaced. These defects could be traced back to the manufacturing process combining the PP with the CGM-IS. These defective devices were excluded from the survival rate evaluation as they were never inserted into participants.

**Figure 4.**
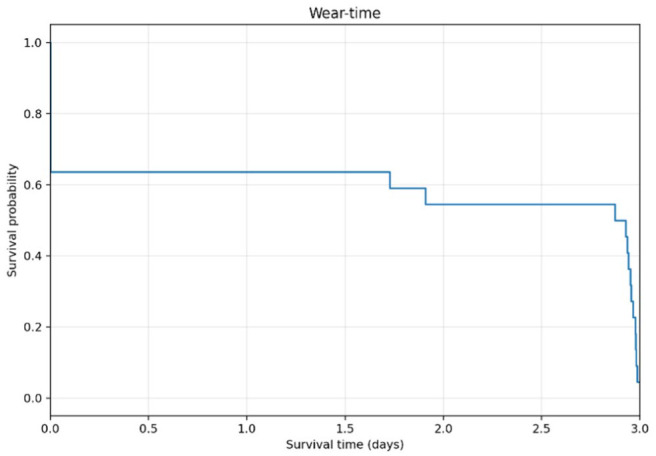
Device survival for participants in the main study phase.

A total of 26 devices were inserted into participants. Seventeen devices in total from 17 participants (three devices from three participants from the pilot phase and 14 devices from 15 participants from the main phase of the study) provided sensor and insulin delivery data for analysis. Fourteen participants from the main phase of the study also provided data for analysis of day 2 insulin delivery. Supplemental Figure S1 provides a flow chart for all devices utilised in the study. There were four devices inserted in three participants in the pilot phase. Participant 1 had a spurious CGM signal and the device was replaced before commencement of the meal test. In the main phase of the study, 22 devices were inserted in 15 participants. Eight devices failed in four participants at insertion on day 1 providing no meaningful sensor data for analysis. All failures were the result of spurious or absent CGM currents. None were due to a failure of insulin delivery. Participant 14 had 4/4 devices fail post-insertion and no meaningful data were obtained from this participant due to a low or absent sensor signal. Further insertions were not performed following four failed attempts. There were four additional device failures at insertion across the remaining three participants (5, 10 and 12) who had their devices replaced and continued with the study. Mean (SD) duration of device survival of all 22 devices used in the main phase of the study was 42 hours and 42 minutes (33 hours and 56 minutes). The 14 devices in the main phase that provided sensor information lasted a mean of 66 hours and 58 minutes (9 hours and 59 minutes). For participant SM04, after two failed insertions, the third device inserted provided sensor data for analysis and failed at 1.7 days when the wires between the pump and the transmitter broke due to improper taping of the wires. The device for SM-05 completed the study without incident and the shorter duration of 1.9 days is explained by the fact that the two preceding failed devices consumed part of the study time allotted to the patient with no sensor data available for the first meal test. The other 12 devices were worn for the entire three-day protocol and removed at the end of the study. Device survival over time for all 22 devices inserted into participants for the main phase is shown in Figure 4. Individual data for device survival are provided in the supplementary appendix (S2).

### Glucose Sensor Performance

Glucose sensor performance is reported for the combined cohort of 17 participants, which included those in the pilot phase (n = 3) and the main phase (n = 14).

**Figure 5.**
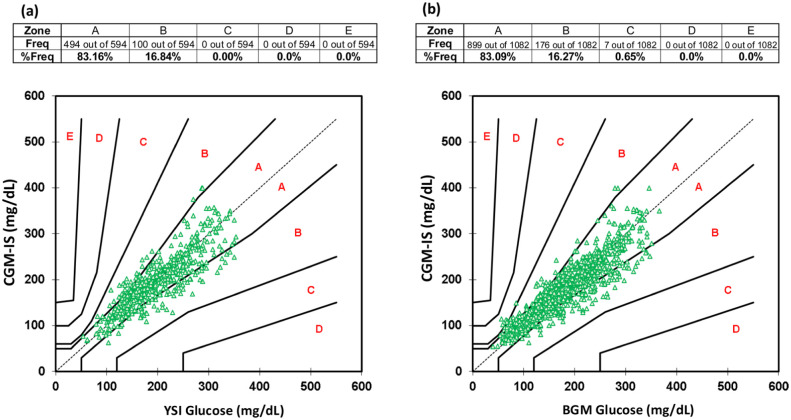
Consensus Error Grid for (a) PP-CGM-IS vs YSI, and (b) PP-CGM-IS vs BGM.

Benchmarked against YSI, aggregated MARD for the glucose excursions during each meal test for 17 participants was 11.6 ± 10.2% with a total of 594 data points (Table 2). The CEG plot for CGM-IS against YSI revealed that 83.16% values fell within zone A and 100% fell within zones A + B (Figure 5a).

**Table 2.** Mean Absolute Difference (MAD)/Mean Absolute Relative Difference (MARD) for Participants.

	CGM-IS vs YSI		CGM-IS vs BGM		
Participant ID	MARD (%)	n	MARD (%)	BG average (mg/dL)	n
**SMART-01**	19.6	18	19.1	197.4	23
**SMART-02**	15.2	21	14.9	175.1	29
**SMART-03**	12.4	21	12.0	202	24
**SMART-04**	10.8	20	15.3	169	49
**SMART-05**	14.4	21	15.0	145.2	40
**SMART-06**	13.1	42	13.9	179.8	77
**SMART-07**	7.7	41	10.4	242.6	71
**SMART-08**	12.4	40	10.6	161	80
**SMART-09**	7.0	39	7.6	177	78
**SMART-10**	13.2	41	12.1	161.6	100
**SMART-11**	14.1	42	11.0	198.8	78
**SMART-12**	8.2	41	7.6	199.3	68
**SMART-13**	15.6	41	13.4	174.2	74
**SMART-14** ^a^	N/A	N/A	N/A	N/A	N/A
**SMART-15**	12.1	41	12.3	216	72
**SMART-16**	9.2	41	15.5	164.6	74
**SMART-17** ^b^	N/A	N/A	N/A	N/A	N/A
**SMART-18**	7.8	42	9.7	165	66
**SMART-19**	11.7	42	11.9	166.8	79
**Total**	**11.6**	**594**	**12.0**	**182.1**	**1082**
**SD (%)**	10.2		11.1		

a No sensor signal obtained after four device insertions.

b Participant withdrew prior to device insertion.

There were 1082 paired device and BGM readings obtained. Benchmarked against the study BGM, MARD during the meal tests was 12.0 ± 11.1% (Table 2). Consensus error grid with metered glucose readings as the comparator revealed 83.18% of readings in zone A and 99.35% of readings within zones A + B (Figure 5b). Mean absolute relative difference analysis for each day and meal test is available in the supplementary appendix (S3).

### Insulin Delivery and Glucose Levels

Insulin delivery and glucose levels are reported for the main phase only. Of the 15 participants who commenced the main phase of the study, no data were available for participant 14 as despite repeated insertions a functioning device was unable to be successfully implemented. Data uploaded from the commercial CGM for participant 10 was unable to be accessed from the web-based platform. Therefore, 14 complete insulin delivery and 13 complete CGM data sets were available. One participant used their insulin pump in manual mode during run-in, the remainder utilised AID. Time in range was 80.3 ± 14.0% at run-in vs 62.2 ± 13.7% on day 2 with the commercial CGM (difference = –18.1%, *P* = .0001). Mean glucose at run-in was 140.4 ± 19.8 versus 165.6 ± 19.8 mg/dL on day 2 (difference = 25.2 mg/dL, *P* = .0002). Total daily insulin dose at run-in was 42.7 ± 16.9 versus 37.3 ± 13.5 units/day (difference = –5.4 units/day, *P* = .045) (Table 3). Time for insulin bolus delivery was calculated using 28 meal boluses across the two meal tests during the main phase. Mean duration was 8 minutes, 26 seconds ± 0.01 minutes and mean insulin bolus was 7.6 ± 2.3 units. There were no insulin delivery failures.

**Table 3.** Time in Range, Mean Blood Glucose, and Total Daily Insulin Dose at Baseline Versus Day 2.

	Baseline	Day 2	Difference	*P* value
CGM				
TIR (%)^a^	80.3 ± 14.0	62.2 ± 13.7	–18.1 (–22.6)	.0001
Mean glucose (mg/dL)^a^	140.4 ± 19.8	165.6 ± 19.8	25.2 (17.9)	.0002
**Insulin delivery**				
TDD (units/day)^b^	42.7 ± 16.9	37.3 ± 13.5	–5.4 (–12.7)	.045
Basal (units/day)^b^	21.6 ± 13.9	18.0 ± 6.3	–3.6 (–16.8)	.21
Bolus (units/day)^b^	21.1 ± 7.7	19.3 ± 9.3	–1.8 (–8.6)	.29

All results expressed as mean (SD).

Abbreviations: basal, total daily basal insulin; bolus, total daily bolus insulin; Mean glucose, mean CGM glucose; TDD, total daily insulin dose.

a Data available for n = 13 participants; TIR, CGM time in range.

b Data available for n = 14 participants.

### Adverse Events

There were no serious adverse events. Notably there were no episodes of diabetic ketoacidosis (DKA), hyperglycaemia with ketones or other device-related adverse events. Despite being a prototype, the device was well tolerated, and most participants had no or very slight oedema (100%) or erythema (66.7%).

## Discussion

To the best of our knowledge, for the first time, this proof-of-concept single-arm, non-randomised study has demonstrated that a PP integrating glucose sensing at the site of insulin delivery in a single-insertion device is feasible. While further refinement is required, the approach has the potential to substantially reduce the physical and psychological burden imposed upon the user while enhancing functionality and reducing the environmental footprint of these devices.

Although feasibility was established using these early-stage prototypes of a combined device our experience suggests a significant need for refinement in the manufacture, transport, and the mechanics in inserting the device with 12/38 devices unusable and a further 9/38 of the devices failing immediately or shortly following insertion. While these limitations are important, they are addressable in later product development and require further investigation. Those 14 devices that were successfully inserted in the main phase lasted almost 67 hours supporting feasibility of the approach. Insulin delivery appeared robust. The higher glucose levels observed during the in-hotel phase in comparison with run-in was associated with a decrease in insulin dosed, which could be attributed to differing conditions of insulin delivery (manual dosing of insulin vs automated dosing) and activity (routine vs sedentary with restricted movements and a modified diet). In addition, the estimated total daily insulin requirements for the study were determined manually and often conservatively dosed lower than run-in for safety, however, requirements were adjusted during the study at the investigators’ discretion. Importantly, there were no issues with ketosis or episodes of DKA.

Performance of the sensor component of the PP-CGM-IS revealing MARDs at 11.6% and 12.0% when benchmarked against YSI and BGM, respectively, was promising. However, it should be noted that the glucose prediction algorithm was developed retrospectively. While not part of the formal analysis, the investigational sensor performance was comparable with the commercially available, factory-calibrated CGM (10.4% and 11.2%, respectively) which was worn simultaneously during the study when calculated using the same benchmarks. The insertion site of the commercially available sensor was well away from the insulin infusion site and it may be assumed that the commercial sensor’s function was not subject to interference by the adjacent delivery of insulin. Sensor issues that occurred post-insertion were responsible for all premature system removals prior to the end of the study. This differs from observations with our earlier study where there were no premature removals due to sensor function and sensor accuracy during the meal test on day 4 was 9.8%^6^ suggesting that either the manufacturing process of incorporating the CGM-IS as part of the PP or the insertion process may have impacted both sensor durability and accuracy.

Finally, the PP itself performed well with no issues related to the pumping mechanism and its reservoir. The devices were well tolerated and there were no infections or skin reactions.

Having determined feasibility of combining a PP with a single insertion that combined glucose sensing with an insulin delivery cannula, further development is required to more completely integrate CGM-IS function into the PP. The iteration tested in the current study incorporated an external transmitter, which relayed sensor data to a handset. Moving forward, it would be expected that this component would be incorporated as part of the PP itself. Moreover, almost all commercially available insulin pumps automate insulin dosing. Insulin dosing was manually determined as we did not wish to compromise participant safety in this proof-of-concept study. The ultimate aim would be to incorporate an automatic glycaemic control algorithm for AID within the body of the PP-CGM-IS, which would receive glucose data directly from the glucose sensing component of the CGM-IS. The PP-CGM-IS studied utilised a re-usable cover, housing the pump battery and electronics, which aims to further minimise the environmental footprint.

There were limitations in our assessment of insulin delivery with differing insulin requirements and mode of insulin dosing (manual vs automated) during run-in and following investigational device provision. Aligning these would have permitted a more rigorous assessment of the integrity of insulin delivery. However, safety was prioritised, which mandated that the participants spent the study under close observation in a hotel and that the investigation team operate the pump itself without AID functionality. Strengths included the meal tests designed to provide a wide range of glucose readings enabling a meaningful assessment of sensor accuracy.

## Conclusion

In conclusion, we have for the first time demonstrated feasibility of a PP-CGM-IS in persons living with T1D. These devices may be more acceptable to people with this condition than conventional tubed pumps or PPs with a separate sensor. Further research will be aimed at refining the device, its manufacture with an improved insertion process, greater sensor accuracy with a real-time algorithm and sensor robustness. Ultimately, the device should also include AID functionality, which represents the current standard of care.

## Supplemental Material

sj-pptx-1-dst-10.1177_19322968261436412 – Supplemental material for First in Human Feasibility Study of an Insulin Patch Pump Combined With CGM-Insulin Delivery Cannula Supplemental material, sj-pptx-1-dst-10.1177_19322968261436412 for First in Human Feasibility Study of an Insulin Patch Pump Combined With CGM-Insulin Delivery Cannula by Hannah Cunningham, Hanna Jones, Steve Flint, Emma Netzer, Ralph Dutt-Ballerstadt, Peter Eckenberg, Varuni Obeyesekere, Katrin Ellen Brown, Catriona Sims, Solomon Reid, Huan-Ping Wu, Thomas Seidl, Marcel Both, Adrian Auderset, Daniel Eymann, Tamara Lodico, Michael Schoemaker, Yee Wen Kong and David Norman O’Neal in Journal of Diabetes Science and Technology
