# Supplementary material for: First in Human Feasibility Study of an Insulin Patch Pump Combined With CGM-Insulin Delivery Cannula
Source: J Diabetes Sci Technol. 2026 Apr 9:19322968261436412. Online ahead of print. doi: 10.1177/19322968261436412 (PMC13065641; doi:10.1177/19322968261436412)
Supplement: sj-pptx-1-dst-10.1177_19322968261436412 – Supplemental material for First in Human Feasibility Study of an Insulin Patch Pump Combined With CGM-Insulin Delivery Cannula [file sj-pptx-1-dst-10.1177_19322968261436412.pptx]

## Slide 1
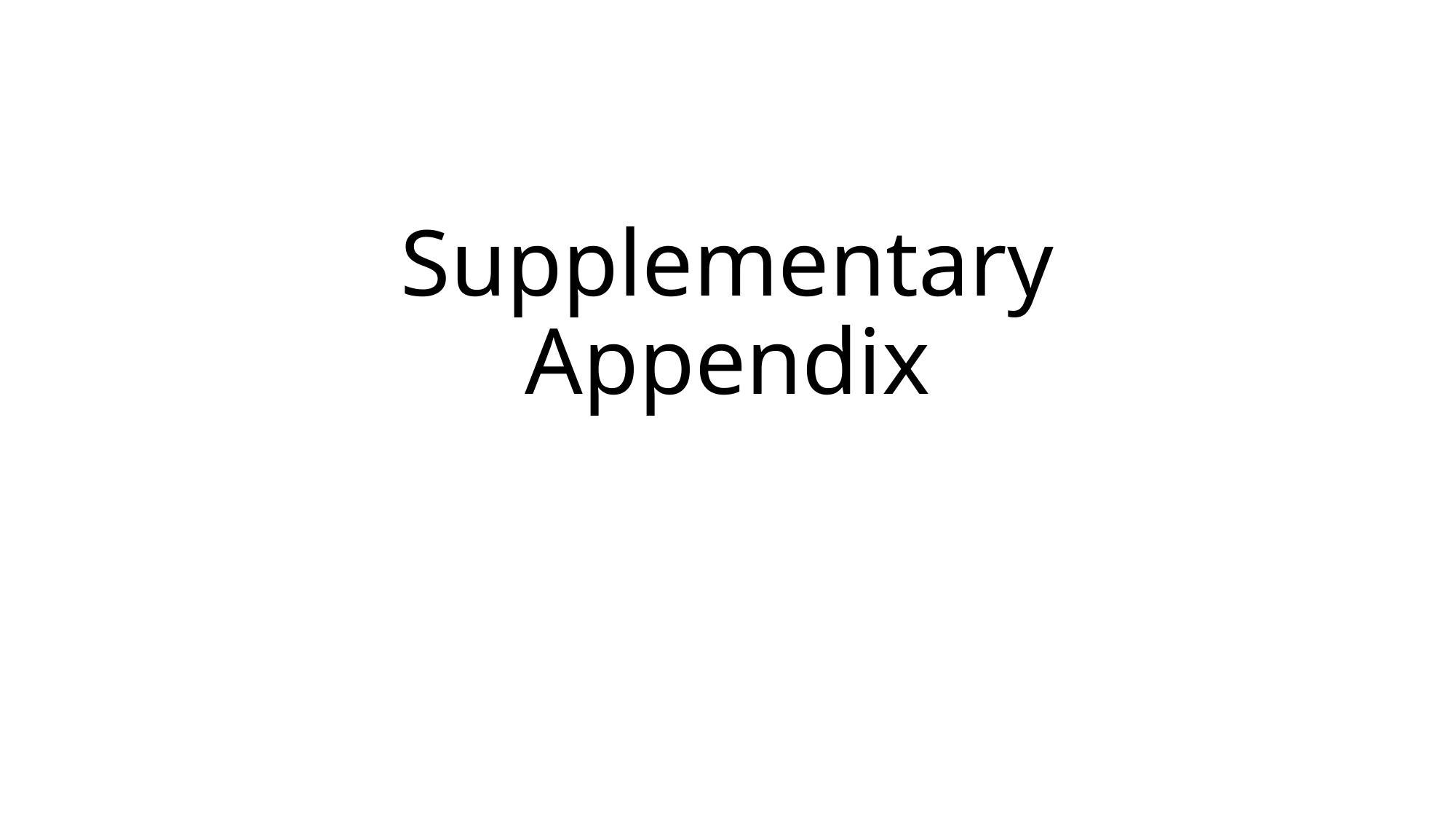

# Supplementary Appendix

## Slide 2
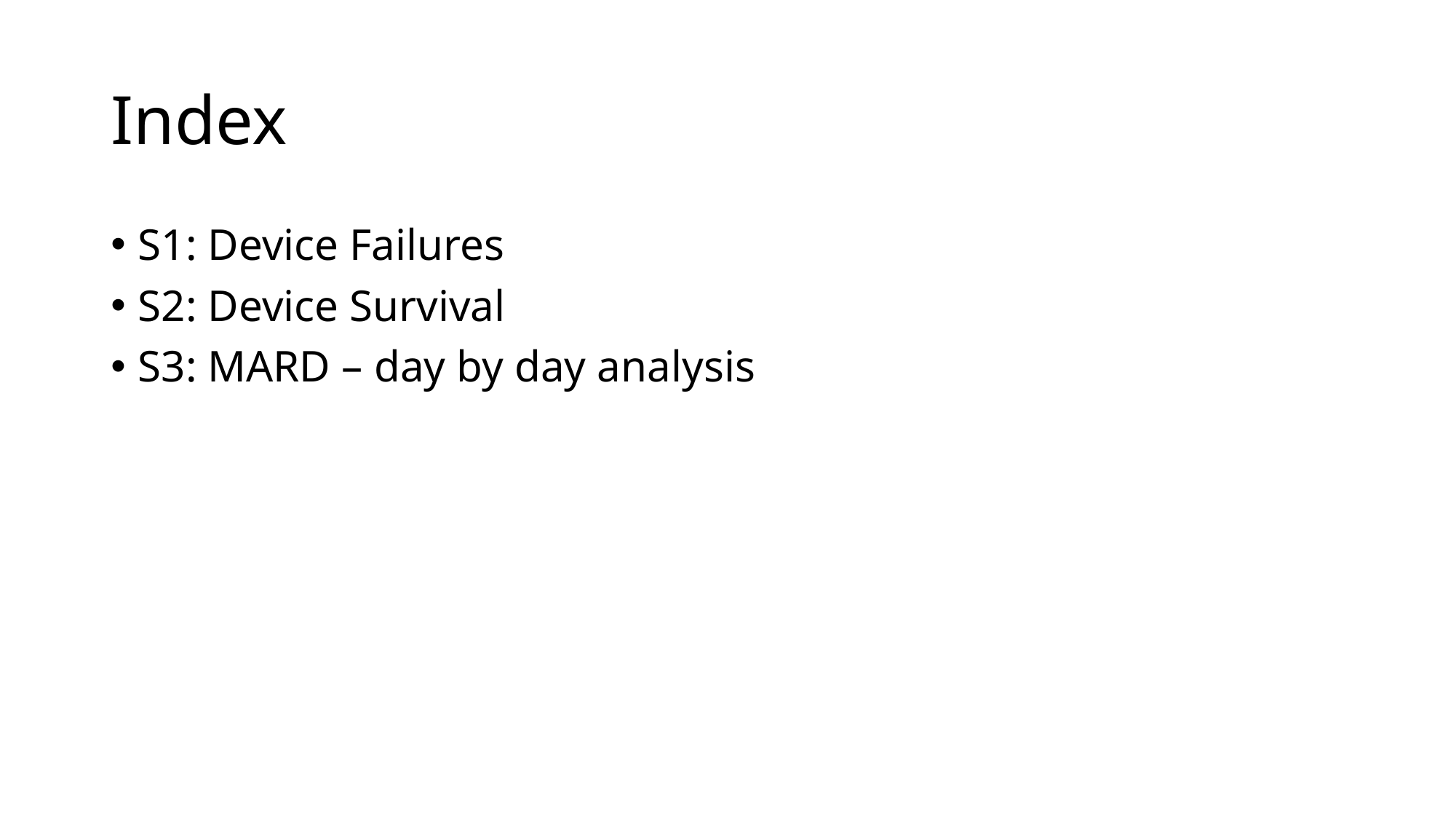

# Index
S1: Device Failures
S2: Device Survival
S3: MARD – day by day analysis

## Slide 3
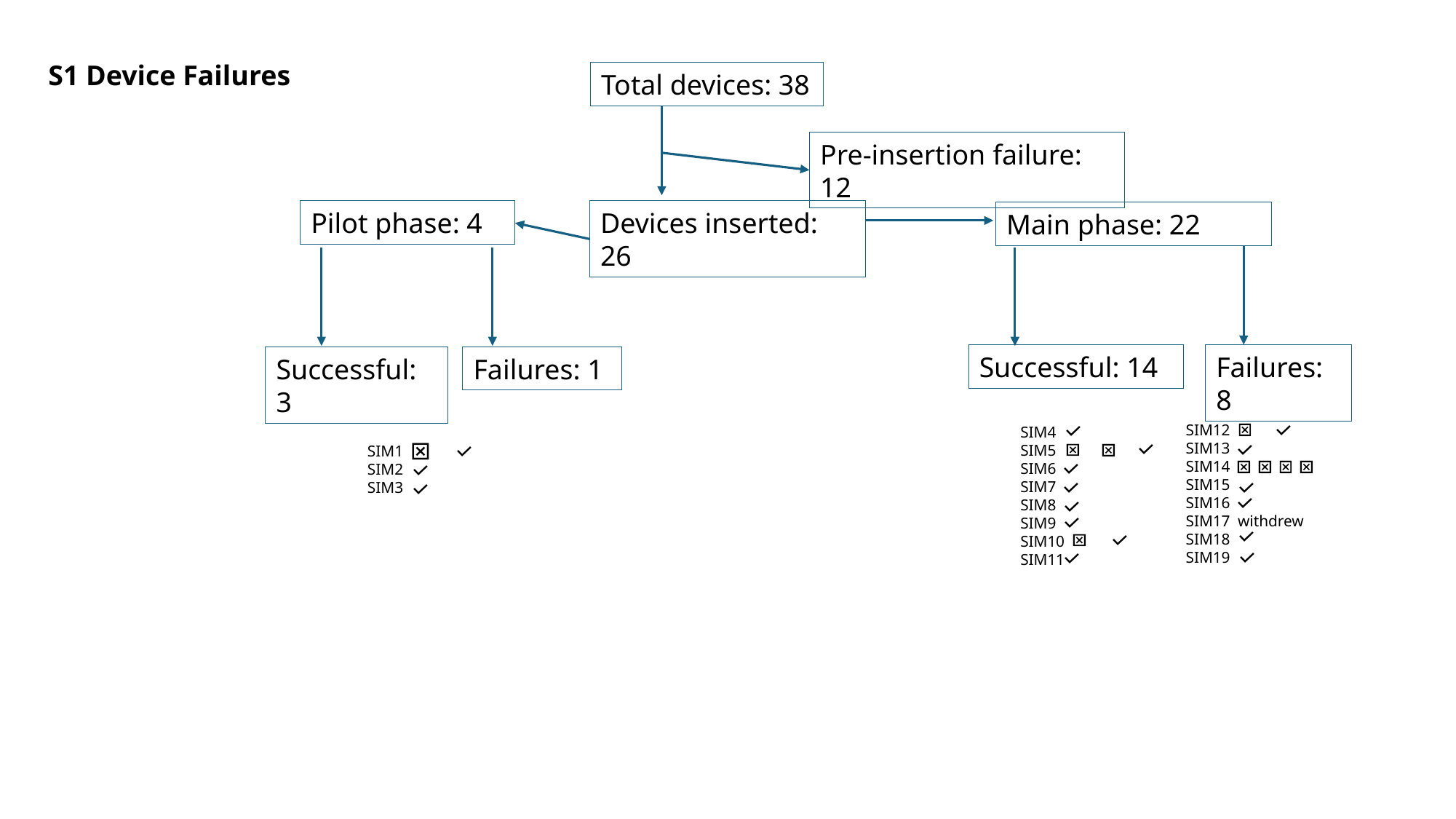

# S1 Device Failures
Total devices: 38
Pre-insertion failure: 12
Pilot phase: 4
Devices inserted: 26
Main phase: 22
Successful: 14
Failures: 8
Failures: 1
Successful: 3
SIM12
SIM13
SIM14
SIM15
SIM16
SIM17 withdrew
SIM18
SIM19
SIM4
SIM5
SIM6
SIM7
SIM8
SIM9
SIM10
SIM11
SIM1
SIM2
SIM3

## Slide 4
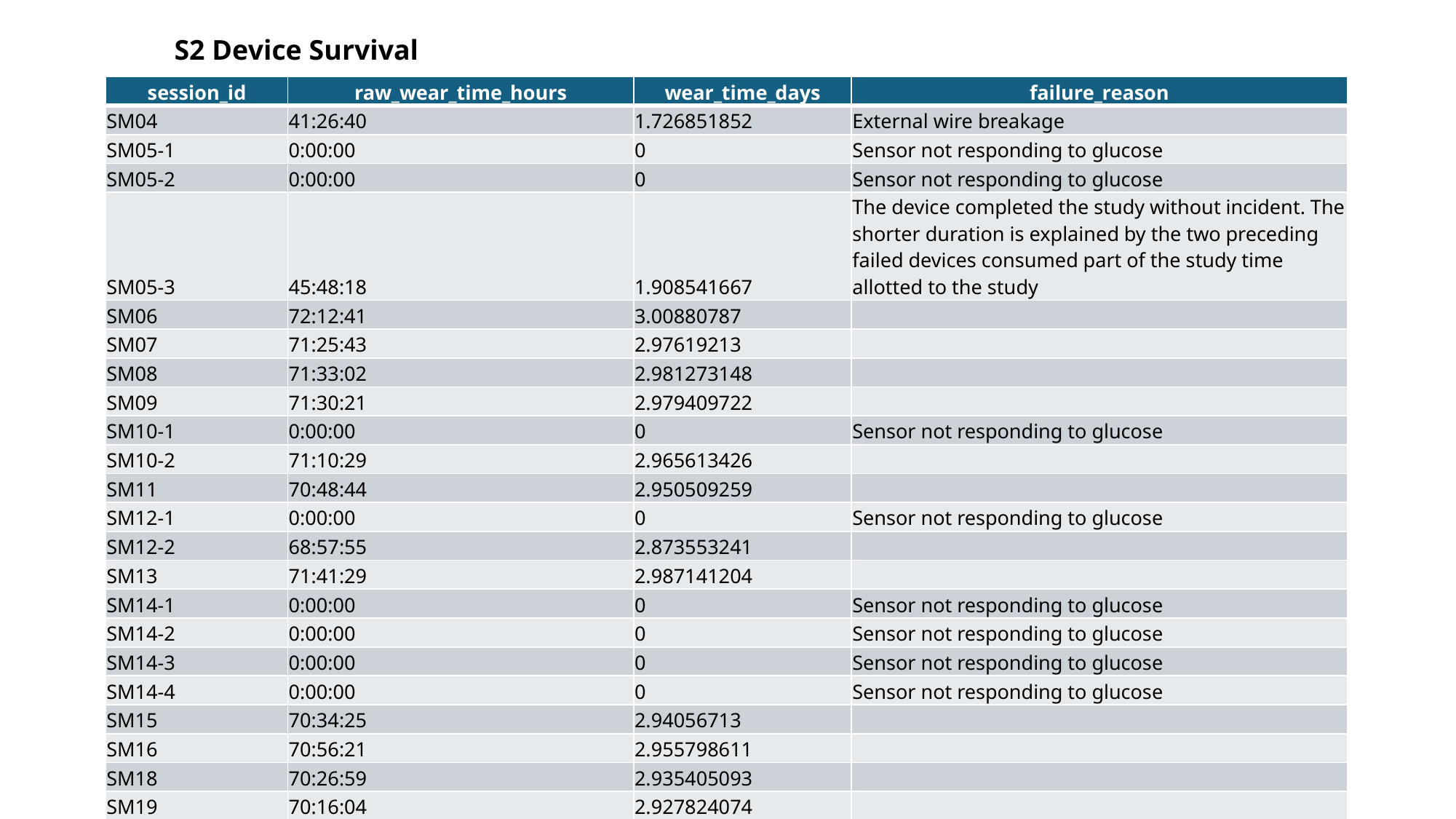

# S2 Device Survival
| session\_id | raw\_wear\_time\_hours | wear\_time\_days | failure\_reason |
| --- | --- | --- | --- |
| SM04 | 41:26:40 | 1.726851852 | External wire breakage |
| SM05-1 | 0:00:00 | 0 | Sensor not responding to glucose |
| SM05-2 | 0:00:00 | 0 | Sensor not responding to glucose |
| SM05-3 | 45:48:18 | 1.908541667 | The device completed the study without incident. The shorter duration is explained by the two preceding failed devices consumed part of the study time allotted to the study |
| SM06 | 72:12:41 | 3.00880787 | |
| SM07 | 71:25:43 | 2.97619213 | |
| SM08 | 71:33:02 | 2.981273148 | |
| SM09 | 71:30:21 | 2.979409722 | |
| SM10-1 | 0:00:00 | 0 | Sensor not responding to glucose |
| SM10-2 | 71:10:29 | 2.965613426 | |
| SM11 | 70:48:44 | 2.950509259 | |
| SM12-1 | 0:00:00 | 0 | Sensor not responding to glucose |
| SM12-2 | 68:57:55 | 2.873553241 | |
| SM13 | 71:41:29 | 2.987141204 | |
| SM14-1 | 0:00:00 | 0 | Sensor not responding to glucose |
| SM14-2 | 0:00:00 | 0 | Sensor not responding to glucose |
| SM14-3 | 0:00:00 | 0 | Sensor not responding to glucose |
| SM14-4 | 0:00:00 | 0 | Sensor not responding to glucose |
| SM15 | 70:34:25 | 2.94056713 | |
| SM16 | 70:56:21 | 2.955798611 | |
| SM18 | 70:26:59 | 2.935405093 | |
| SM19 | 70:16:04 | 2.927824074 | |

## Slide 5
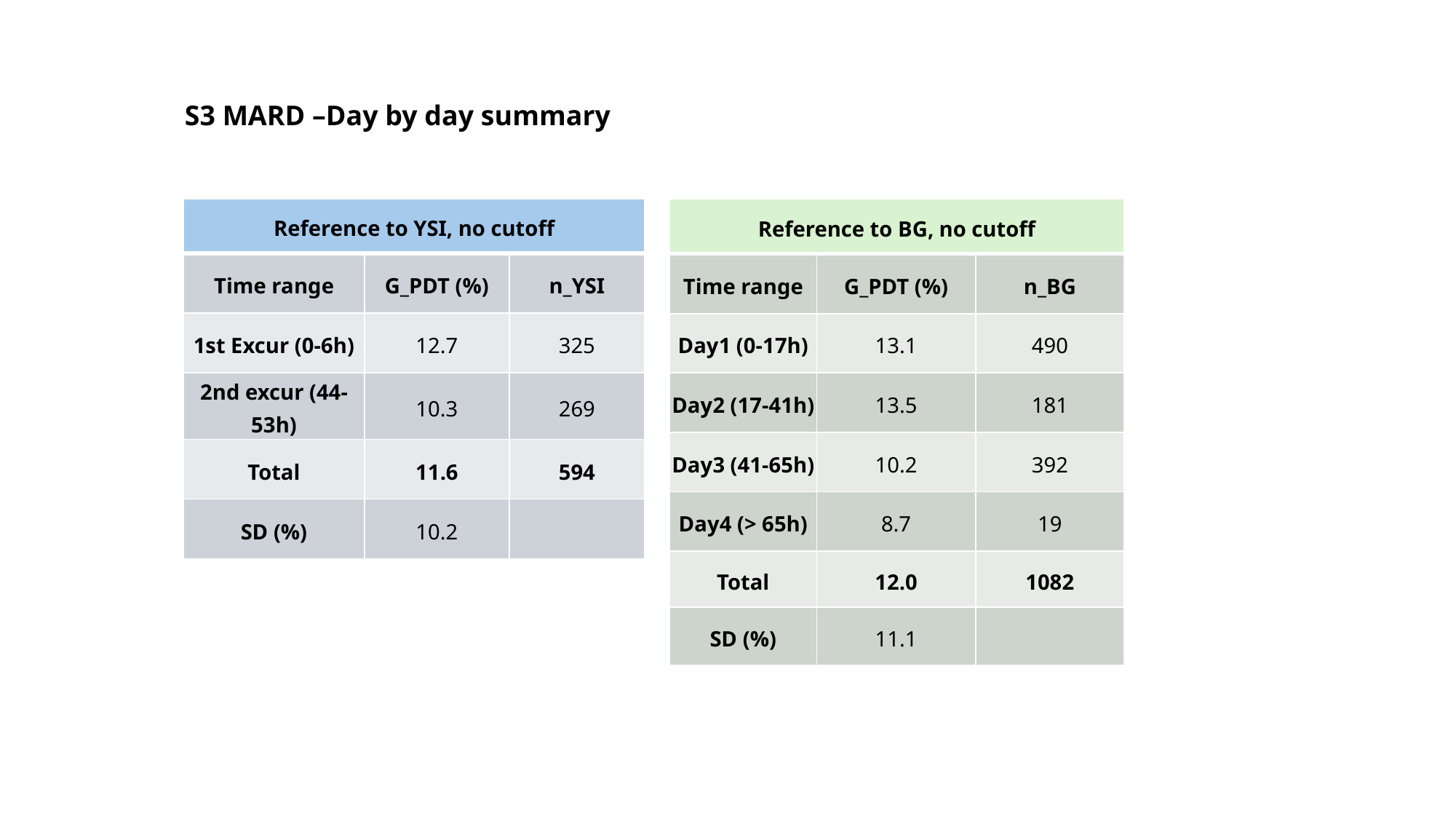

# S3 MARD –Day by day summary
| Reference to YSI, no cutoff | | |
| --- | --- | --- |
| Time range | G\_PDT (%) | n\_YSI |
| 1st Excur (0-6h) | 12.7 | 325 |
| 2nd excur (44-53h) | 10.3 | 269 |
| Total | 11.6 | 594 |
| SD (%) | 10.2 | |
| Reference to BG, no cutoff | | |
| --- | --- | --- |
| Time range | G\_PDT (%) | n\_BG |
| Day1 (0-17h) | 13.1 | 490 |
| Day2 (17-41h) | 13.5 | 181 |
| Day3 (41-65h) | 10.2 | 392 |
| Day4 (> 65h) | 8.7 | 19 |
| Total | 12.0 | 1082 |
| SD (%) | 11.1 | |
